# miRNAs associated with prostate cancer risk and progression

**DOI:** 10.1186/s12894-017-0206-6

**Published:** 2017-03-20

**Authors:** Hung N. Luu, Hui-Yi Lin, Karina Dalsgaard Sørensen, Olorunseun O. Ogunwobi, Nagi Kumar, Ganna Chornokur, Catherine Phelan, Dominique Jones, LaCreis Kidd, Jyotsna Batra, Kosj Yamoah, Anders Berglund, Robert J. Rounbehler, Mihi Yang, Sang Haak Lee, Nahyeon Kang, Seung Joon Kim, Jong Y. Park, Giuliano Di Pietro

**Affiliations:** 1 Division of Epidemiology, Department of Medicine, Vanderbilt Epidemiology Center, Vanderbilt-Ingram Cancer Center, Vanderbilt University School of Medicine, Nashville, TN USA; 2 Department of Epidemiology and Biostatistics, College of Public Health, University of South Florida, Tampa, FL USA; 3 Biostatistics Program, School of Public Health, Louisiana State University Health Sciences Center, New Orleans, LA 70112 USA; 4 Department of Molecular Medicine, Aarhus University Hospital, Aarhus, Denmark; 5 Department of Biological Sciences, Hunter College of The City University of New York, New York, NY 10065 USA; 6 Department of Cancer Epidemiology, H. Lee Moffitt Cancer Center and Research Institute, Tampa, FL 33612 USA; 7 Department of Pharmacology and Toxicology, James Brown Cancer Center, University of Louisville School of Medicine, Louisville, KY 40202 USA; 8 Australian Prostate Cancer Research Centre-QLD, Institute of Health and Biomedical Innovation and School of Biomedical Sciences, Translational Research Institute, Queensland University of Technology, Brisbane, Australia; 9 Department of Radiation Oncology, H. Lee Moffitt Cancer Center and Research Institute, Tampa, FL 33612 USA; 10 Department of Biostatistics and Bioinformatics, H. Lee Moffitt Cancer Center and Research Institute, Tampa, FL 33612 USA; 11 Department of Tumor Biology, H. Lee Moffitt Cancer Center and Research Institute, Tampa, FL 33612 USA; 12 Research Center for Cell Fate Control, College of Pharmacy, Sookmyoung Women’s University, Seoul, Republic of Korea; 13 Department of Internal Medicine, The Cancer Research Institute, College of Medicine, The Catholic University of Korea, Seoul, Republic of Korea; 14 Department of Pharmacy, Federal University of Sergipe, Rodovia Marechal Rodon, Jardim Rosa Elze, Sao Cristóvão, Brazil

## Abstract

Prostate cancer is the most common malignancy among men in the US. Though considerable improvement in the diagnosis of prostate cancer has been achieved in the past decade, predicting disease outcome remains a major clinical challenge. Recent expression profiling studies in prostate cancer suggest microRNAs (miRNAs) may serve as potential biomarkers for prostate cancer risk and disease progression. miRNAs comprise a large family of about 22-nucleotide-long non-protein coding RNAs, regulate gene expression post-transcriptionally and participate in the regulation of numerous cellular processes. In this review, we discuss the current status of miRNA in studies evaluating the disease progression of prostate cancer. The discussion highlights key findings from previous studies, which reported the role of miRNAs in risk and progression of prostate cancer, providing an understanding of the influence of miRNA on prostate cancer. Our review indicates that somewhat consistent results exist between these studies and reports on several prostate cancer related miRNAs. Present promising candidates are miR-1, −21, 106b, 141, −145, −205, −221, and −375, which are the most frequently studied and seem to be the most promising for diagnosis and prognosis for prostate cancer. Nevertheless, the findings from previous studies suggest miRNAs may play an important role in the risk and progression of prostate cancer as promising biomarkers.

## Background

Prostate cancer (PCa) is the most common malignancy among men in the US. In 2016, there were approximately 180,890 new cases and 26,120 PCa related deaths [1]. Over the past two decades, PCa detection with serum prostate specific antigen (PSA/KLK3) has led to a significant increase in the detection of low-grade PCa (Gleason < 6), a disease that has been shown to pose little risk of either metastatic spread or death. Active surveillance has thus evolved as a recommended management strategy for men with low grade disease. On the other hand, in 10 to 15% of cases, the cancer is aggressive and advances beyond the prostate, sometimes turning lethal, characterized by recurrence and early metastasis through the bloodstream and lymphatic system. Moreover, distant disease is associated with a low 5-year survival rate of 28% (2014–2015 ACS Cancer Treatment and Survivorship Facts and Figures). It should also be noted that racial differences may influence the diagnosis and also the progression and PCa treatment [2].

The most important clinical challenge is to distinguish men who have a potentially lethal form of PCa from those with an indolent disease [3]. Given the significant morbidity associated with treatment interventions, active surveillance could help identify men with aggressive disease who would benefit from intensive treatment compared to those in which conservative measures would suffice. Currently, the stratification of PCa patients is guided by the PSA kinetics, clinical stage, and the grade of tumor (Gleason score). Although these parameters are still clinically useful, they have limitations in detecting cases, predicting disease outcomes and guiding clinical management decisions. For example, the sensitivity of PSA with standard cut-off of 4 ng/mL is only 20.5% while the specificity is 93.6% [4]. Thus new biomarkers are urgently needed to improve existing diagnostic, prognostic and treatment management plans. Results from numerous reports suggest a critical role of microRNAs (miRNAs) in PCa progression (reviewed in [5]). These studies reveal expression of miRNAs are dysregulated in prostate tumor and indicated miRNAs may serve as promising PCa risk stratification biomarkers [5].

miRNAs comprise a large family of ~22-nucleotide-long non-protein coding RNAs that regulate gene expression post-transcriptionally by both destabilizing mRNA transcripts and repressing their translation. Since the discovery of the first miRNA (lin-4) in 1993 [6], many other miRNAs have been identified [5, 7]. Reportedly, miRNAs normally regulate cellular processes such as development and differentiation in multicellular organisms [8, 9]. Subsequent studies found that expression of miRNAs are significantly associated with various human cancers, including prostate cancer. For example, miR-143, miR-145, and miR-375 were identified as biomarkers with a high-level sensitivity and specificity in PCa detection [10], albeit unequivocally [5].

Most miRNAs bind at the 3′ untranslated regions (3′ UTR), 5′ UTR and coding mRNA regions, which leads to mRNA degradation or translational inhibition [11–13]. miRNAs regulate the activity of more than 60% of all human gene products [9, 14, 15]. Biological functional studies suggest miRNAs are involved in the regulation a plethora of cellular processes, including those essential to tumorigenesis [14]. Each miRNA may regulate hundreds of genes with varying specificities through base pairing to mRNAs [16]. Moreover, a certain gene may serve as a targeted by multiple miRNAs via different binding sites [17, 18]. Therefore, a miRNA can play a role in multiple biological cell signaling pathways by regulating the expressions and functions of their target genes [19]. Currently, there are over 1,800 human miRNAs identified. Many of them are expressed in a tissue-specific manner and changes in the expression of certain miRNAs are associated with various cancers including PCa (miRBase Release 21, www.mirbase.org) [14].

In this review, we will provide overview information on the current status of miRNA studies in PCa. The discussion will highlight key findings from human studies, examine the expression of miRNA in prostate tumor tissues with a view to understand the influence of miRNAs on prostate carcinogenesis or possibly the modulation of the clinical course of the disease. We will also review studies comparing the expression of miRNAs in different biological states of PCa via tumor tissue and biological fluid relative to controls. The review will conclude with a discussion of the therapeutic potential of miRNAs in PCa and on future directions of miRNA research in PCa.

## miRNA and prostate cancer

miRNAs affect gene expression significantly and their dysregulation may cause tumorigenesis and its progression. The first report of miRNA’s role in human cancer came from a study that characterized chromosome 13q14 in chronic lymphocytic leukemia (CLL) [20]. Since the first report of aberrant miRNA expression in CLL, the dysregulation of a number of miRNAs is closely associated with the development and progression of various cancers. Furthermore, about 50% of miRNA sequences are located in cancer-related regions (including fragile sites, breakpoint regions, viral integration sites and regions frequently linked with loss of heterozygosity or amplification) in the human genome, suggesting a role of miRNAs in cancer [21]. Moreover, recent studies indicated the dysregulation of miRNA profiles in PCa based on high-throughput methodologies (i.e., microarrays, RNA-Seq, proteomic arrays).

Further, miRNA expression profiles may help to distinguish between normal and tumor tissues [22–28]. miRNA profiles appear to have a better accuracy to differentiate between tumor and normal tissues when compared to mRNA profiling [29]. Expression profiles of miRNAs often correspond with clinic-pathological parameters and predict patient outcome or response to treatment [22, 30, 31]. These observations underscore the potential of miRNAs as new diagnostic or prognostic biomarkers. Additionally, the oncogenic or tumor-suppressive functions of miRNAs [24, 32] suggested that miRNAs may be new candidates for cancer drug treatment. Beyond that, miRNAs has also been shown to be more stable than the mRNA and are therefore more suitable to be measured in formalin-fixed paraffin-embedded (FFPE) samples [33, 34]. The stability of miRNAs also makes miRNAs better candidates for blood-based markers [35, 36].

Dysregulation of miRNA in cancer could be caused by several genomic aberrations. These genomic anomalies may include, chromosomal modification, such as translocation, change of transcriptional factors and aberrant expression, epigenetic alterations, and changes in miRNA processing [22]. Several studies reveal miR-155 is induced at the transcriptional level by transforming growth factor β (TGFB1)/Smad, nuclear factor-κB (NFKB1) and activator protein-1 (AP1) family transcription factors (JUN/JUNB/JUND/FOS/FOSB) through direct interaction with the miR-155 promoter [37–39].

## miRNAs as oncogenes and tumor suppressors

miRNAs are divided into two types, oncogenes or tumor suppressors based upon whether their targets. Several miRNAs have been characterized as oncogenes and tumor suppressors in PCa. miRNAs inhibit the expression of tumor suppressor genes via their up-regulation in tumor cells, and are commonly termed oncogenic miRNAs or onco-miRs. The oncogenic miRNAs in PCa may enhance proliferative function by down-regulation of cell-cycle dependent kinase inhibitors p21 and p27 [40–42], as well as members of the E2F family of transcription factors [40, 43]. For example, miR-17-92 cluster contains six miRNAs (miR-17, −18a, −19a, −19b-1, −20a, and -92a-1) that are over-expressed in PCa [44], particularly miR-20a is over-expressed in prostate tumor tissues [45]. miR-20a decreases apoptosis in PCa PC-3 cells through repression of E2F2 and E2F3 and inhibition of miR-20a by antisense oligonucleotide, inducing the cell death [43]. Others PCa-associated onco-miRs, miR-1, −21, −106b, −125b, −221 and −222 are known.

The expression of miR-1, also considered an onco-miRNA, is inversely related to the WDR6, XPO6 and SMARCA4 expression. However, miR-1 expression correlates positively with TWF1 transcription (PTK9) in PCa cells, suggesting that microRNAs binding may lead to mRNA cellular accumulation and sequestration of the inhibited mRNA. miR-1 also inhibit expression of exporter-6 and protein tyrosine kinase 9 (also referred to as A6/twinfilin) in PCa cells [40]. Chang et al. [46] showed that decreased miR-1 levels was modulate by the activation of EGFR, leading to invasiveness and the involvement of TWIST1 in facilitating metastasis bone in PCa cell lines. These results indicated that EGFR functions as a transcription factor, repressing miR-1, which has an onco-suppressive role [46]. Kojima et al. [47] showed a down-regulation of miR-1, miR-15a, miR-16-1, and miR-133a in PCa tissues, and these negative regulations contributed to the proliferation, migration and invasion in PC3 and DU145 cells. miR-1 and miR-133a may functioned as tumor suppressors that regulate PNP, an oncogenic gene in Pca [47]. Ambs et al. [40] and Martens-Uzunova et al. [48] observed down-regulation in PCa tissues and linked to PCa progression, castration-resistant disease, and metastasis. Therefore, miR-1 was also suggested at candidate prognostic biomarker for Pca [46, 47, 49–53].

The miR-21 is overexpressed and plays an important role in PCa tumorigenesis, stimulating invasion and metastasis [45, 54–58], and induced invasion and motility of prostate tumor cells [59]. Ribas et al. [57] demonstrated androgen-induced Androgen Receptor binding to the miR-21 promoter that showed a direct transcriptional regulation, even after castration. Their results demonstrated that miR-21 contribute to PCa androgen receptor-driven proliferation and was overexpressed in LNCaP and LAPC-4 cells and in human tumors, but not correlate with stage, grade, or PSA [57]. The coordinated action of miR-21 and androgen receptor signaling, suppresses TGFBR2 levels through binding to its 3'UTR, enhancing androgen receptor signaling, thereby exerting its oncogenic effects on prostate tumors, through inhibition the suppressor activity of the tumor by TGFβ pathway [59]. The miR-21 may be implicated in the resistance to apoptosis, in motility and invasion in PCa cells [60]. miR-21 is able to downregulate Cyclin dependent kinase inhibitors expression by targeting the coding region of the gene, being able to attenuate the p57Kip2 action, which is inhibit the cell cycle [59]. An overexpressed miR-21 can lead to a decrease in the levels of RECK (Reversion-inducing cysteine-rich protein with Kazal motifs, a key inhibitor of several metaloproteinases) as well, and consequently cause an increase in the PCa promotion and progression [58].

miR-106b which targets the p21 (CDKN1A) and E2F1, displays an anti-apoptotic role in PCa cells by inhibiting caspase activation. Thus miR-106 suppressed E2F1 and p21/WAF1 protein expression [40]. Ambs et al. [40] and Szczyrba et al. [61] observed over-expression of miR-106b in gene expression profiling of prostate tumors. miR-106b appears to be a PCa oncogene, and was suggested as a candidate prognostic marker for PCa [62–64].

miR-125b is usually was over-expressed in androgen-independent PCa cells, but was downregulated in androgen-dependent LNCaP cells. Ectopic expression of miR-125b in LNCaP cells stimulates growth as a consequence of downregulation of its major target BAK1, a pro-apoptotic gene belonging to the Bcl-2 family [65, 66]. The miR-145 binds to the 3'UTR of MYO6 gene and regulated negatively, resulting in a decrease in myosin VI (involved in cancer-related cell migration), and β-actin in LNCaP cell line [61].

Poliseno et al. [62] has shown that miR-25, miR-93, and miR-106b are up-regulated in PCa and are inversely correlated with PTEN (phosphatase and tensin homolog deleted on chromosome 10, is a tumor suppressor that antagonizes signaling via the phosphatidylinositol-3-kinase-Akt pathway) abundance in prostate cancer. The authors also observed that miR-22 transformed MEFs (mouse embryonic fibroblasts) when combined with c-MYC (a regulatory gene encoding a transcription factor), as well as the miR-106b-25 cluster when combined with c-MYC or when combined with MCM7 (mini-chromosome maintenance protein 7). The expression of MCM7 correlated with tumor progression [62].

Hudson et al. [49] have observed that miR-32, miR-106b-25, and miR-375 are up-regulated in primary tumor tissues and miR-106b-25 showed an additional increased in metastatic lesions especially in bones. On the other hand, miR-101 was down-regulated in tumors and metastases. The caspase-7, a novel candidate target of miR-106b-25, and its inhibition may be important in anti-apoptotic and oncogenic functions, promote cell cycle progression and hyper-proliferation, and miR-106b-25 can also alter the cell adhesion properties in PCa. miR-106b-25 negatively regulates Fas-activated kinase involved in apoptosis. The authors hypothesized that the combined inhibition of pro-apoptotic E2F1, Bim (apoptosis regulator), Fas-activated kinase (serine/threonine transferase kinase/transferase), caspase-7 and PTEN provides miR-106b-25 expressing in cancer cells. In addition, the down-regulation of cell death-inducing integrin-beta 8, which is a target of miR-106b-25, and miR-93, can still support independent growth anchorage [49]. Liang et al. [64] have observed that REST (RE-1 Silencing Transcription Factor) expression decreases with overexpression of miR-93 and miR-106b-25 induced in LNCaP and PC3 cells hypoxia, and elevated expression of pro-neural Phox2a, as well the ASH-1 transcription factors and TH and ChgA NE markers. On the other hand, under conditions of hypoxia, excessive expression of miR-93 led to the destruction of REST [64].

The miR-200c also is upregulation, and it’s targets with ZEB1 and ZEB2, that are transcription factors, that also are able to negatively regulate the miR-200c expression, thus increasing E-cadherin expression [61]. miR-205 has an onco-suppressive function by inhibiting the transition from epithelial to mesenchymal tissue, cell migration, and invasion in the human prostate [48]. Knockdown of miR-221 and miR-222, which are upregulated in the androgen-independent PCa cancer cell line, reduced the clonogenecity and tumorigenicity of androgen-independent PC-3 cells [41, 42]. Ectopic expression of miR-221 and miR-222 enhanced the colony forming potential and in vivo growth of androgen-dependent LNCaP cells by inducing a G1-S shift in the cell cycle [41].

In contrast to onco-miRs, tumor suppressor miRNAs were down-regulated in tumor cells to permit tumor progression. For example, the miR-15a/16-1 cluster located on chromosome 13q14 was frequently deleted in about 50% of PCa tumors [67]. Decreased activity of these two miRNAs resulted in hyperplasia of the prostate in mice and increased expression through intra-tumoral delivery resulted in the regression of PCa xenografts [67, 68]. miR-15a–miR-16-1 cluster controlled cell survival, proliferation and invasion by suppressing cyclin D1 (CCND1) and WNT3A [67]. Similarly, miR-101 expression in PCa was decreased during progression of the disease mainly due to the loss of one or both of its genomic loci. Moreover, miR-101 inhibited the expression and function of EZH2, a member of the polycomb protein group [69].

The tumor-suppressive role of miRNAs in PCa has generally been ascribed to their ability to interfere with cell migration, invasion and pro-apoptotic functions. Ectopic expression of miR-126* significantly reduced the translation of prostein (SLC45A3), which is a prostate-specific protein involved in PCa motility and invasiveness. Presumably, miR-126 binds to the 3′ UTR of prostein mRNA, resulted in reduced invasiveness of LNCaP cells [70]. Similarly, ectopic miR-34a expression induced cell cycle arrest and senescence, inhibited cell growth, and attenuated resistance to the chemotherapeutic agent camptothecin. miR-126 increased chemotherapy sensitivity by inducing apoptosis through suppression of the deacetylase sirtuin (SIRT1) and cyclin dependent kinase 6 (CDK6) and aggressive cellular behavior [71, 72].

Moreover, miR-146a reduced cell proliferation, invasion and metastasis of PC-3 cells to human bone marrow endothelial cell monolayers through the suppression of the Rho-associated protein kinase ROCK1 [73]. Other tumor suppressor-associated miRNAs evaluated in PCa cell models included miR-23b, miR-145, and miR-205. Ectopic expression of miR-23b, and miR-145 in LNCaP cells significantly reduced proliferation [74–77]. Similarly, ectopic expression of miR-205 in PCa cells resulted in reduction of cell locomotion and invasion and downregulation of several oncogenes involved in PCa progression [78].

Although functional studies might define certain miRNAs as onco-miRNAs or tumor suppressor miRNAs, their expression in prostate tumors might not correlate with these classifications. For instance, miR-125b, miR-221, miR-222, miR-373 and miR-520c as onco-miRs in experimental models [41, 42, 65, 66]; however, these miRs were down-regulated in prostate tumor tissue relative to normal prostate tissue [76, 79–81]. miR-373 and miR-520c play a critical role in PCa by inhibiting expression of CD44 RNA [82]. Other miRNAs also defied their given classification as either a tumor suppressor or oncogenic miRNAs. Previous functional studies demonstrated a dual role for miRs-133b and −375 in different PCa cell lines [83–85].

Normally, miR-133b is downregulated as a tumor suppressor miR and miR-375 is upregulated as an oncomiR in PCa tumor tissue relative to normal tissue. However, forced expression of miR-133b increased proliferation in androgen-dependent cells (LNCaP, 22Rv1) [84] while miR-375 decreased proliferation and invasion of androgen-independent PC-3 cells [85]. Thus functional studies performed in experimental models might not perfectly recapitulate clinical disease [86]. In the same way, miR-373 was 1.5 times more prevalent in benign glandular and benign stromal cells, than in the same tissues from PCa patients, as well as miR-520c showed reduced in primary tumors and metastases. Never the less, miR-373 and miR-520c act as pro-invasive agents, mediated by binding with CD44 3'UTR [82].

In summary, miRNAs have an important role in human cancer. More than 50% of miRNA genes are located in cancer-associated regions in the human genome and the expressions of miRNAs are dysregulated in various human cancers, including PCa. Certain miRNAs are upregulated in cancer (onco-miRs), while others are downregulated (tumor suppressors). However, expression profiles and functional studies of miRNAs in PCa cell lines do not always correlate with expression in prostate tumor tissue.

## miRNAs in prostate cancer

Numerous biomarkers have been proposed as promising predictors for risk and/or progression of PCa. However, most biomarkers could not clearly demonstrate their independent clinical potential and, therefore, these biomarkers fail to enhance accuracy of current nomograms. Since the first discovery of miRNAs [6] in 1993, many studies have investigated whether dysregulation of miRNA expression in PCa may correlate with the risk and progression of this disease. These studies reported a number of differentially expressed miRNAs in biological samples from patients with PCa. Tables 1 and 2 summarize results from studies of miRNA expression in PCa.

**Table 1:** Summary of miRNA expression studies on samples from prostate cancer patients

Author year (reference)	Tissue/serum	Outcome	miRNA identified
Risk/Diagnostic			
Ozen, 2008 [79]	Tissue	Risk/Diagnostic	Let-7b-g, 26a-b, 29a-c, 30a-e, 99a-b, 125a-b
Gandellini, 2009 [78]	Tissue	Risk/Diagnostic	miR-205
Mahn 2011 [118]	Tissue/Serum	Diagnostic	let7i, 16, 26a, 195
Wach, 2012 [10]	Tissue	Diagnostic	miR-143, 145, 375
Avgeris, 2013 [101]	Tissue	Diagnostic	miR-145
Srivastava, 2013 [105]	Tissue/Urine	Diagnostic	miR-205, 221, 99b
Tsuchiyama, 2013 [106]	Tissue	Diagnostic	miR-182-5p
Guzel 2015 [107]	Tissue	Diagnostic	miR-361-3p, 133b, 221, 203
Roberts, 2015 [128]	Tissue/Serum	Diagnostic	miR-200b, 200c, 375
Kristensen 2016 [112]	Tissue	Diagnostic	Up: miR-375, 663b, 615-3p, 425-5p, 663a, 182-5p, 183-5p. Down: miR-205-5p, 221-3p, 222-3p, 376c-3p, 136-5p, 455-3p, 455-5p, −154-5p
Mitchell, 2008 [35]	Serum	Diagnostic	miR-141
Lodes, 2009 [137]	Serum	Diagnostic	miR-16, 92a, 103, 107
Moltzahn, 2011 [99]	Serum	Diagnostic	miR-26b, 223, 874, 1274a
Yaman Agaoglu, 2011 [54]	Plasma	Diagnostic	miR-21, 141, 221
Selth, 2012 [114]	Serum	Diagnostic	miR-141, 298, 346, 375
Chen 2012 [119]	Plasma	Diagnostic	let7c, let7e, 30c, 622, 1285
Cheng, 2013 [120]	Serum	Diagnostic	miR-210
Haldrup, 2014 [113]	Serum	Diagnostic	miR-141, 375
Westermann, 2014 [121]	Serum	Diagnostic	miR-141
Kachakova, 2015 [122]	Plasma	Diagnostic	let-7c, 375
Haj-Ahmad, 2014 [126]	Urine	Diagnostic	miR-182-5, 484
Korzeniewski, 2014 [127]	Urine	Diagnostic	miR-483-5p, 1275, 1290
Risk/Progression			
Porkka, 2007 [87]	Tissue	Risk/Progression	Let-7a-d, Let-7f, 19b, 145, 184, 198, 202, 210,
Ambs, 2008 [40]	Tissue	Risk/Progression	miR-1, 32, 101, 106b, 182, 200a, 200b, 494, 520 h
Mercatelli, 2008 [42]	Tissue	Progression	miR-221, 222
Prueitt, 2008 [80]	Tissue	Progression	miR-2, 10, 125b, 224
Tong, 2009 [76]	Tissue	Risk/Progression	miR-23b, 100, 135b, 145, 194, 221, 222
Wang, 2009 [90]	Tissue	Progression	miR-9*, 16, 221, 222, 331-3p, 145, 551a
Szczyrba, 2010 [61]	Tissue	Progression	miR-143
Shaefer, 2010 [81]	Tissue	Risk/Progression	miR-96, 205
Pesta, 2010 [93]	Tissue	Progression	miR-20a
Khan, 2010 [88]	Tissue	Progression	miR-128
Spahn, 2010 [91]	Tissue	Progression	miR-221
Hagman, 2010 [92]	Tissue	Risk/Progression	miR-34c
Brase, 2011 [115]	Tissue/Serum	Progression	miR-141, 375
Barnabas, 2011 [103]	Tissue	Progression	miR-151
Leite, 2011 [138]	Tissue	Progression	let7c, miR-100, miR-145, miR-191
Martens-Uzunova, 2012 [48]	Tissue	Progression	miR-1, 133a, 133b, 143, 143*, 145, 145*, 204, 221, 222
Li, 2012 [55]	Tissue	Progression	miR-21
Tsuchiyama, 2013 [106]	Tissue	Progression	miR-182-5p
Amankwah, 2013 [102]	Tissue	Progression	miR-21
He, 2013 [134]	Tissue	Progression	miR-19a, miR-374b
Kristensen, 2014 [89]	Tissue	Progression	miR-224, 452
Wang, 2014 [124]	Tissue/Serum	Progression	miR-19, miR-345, miR-519c-5p
Dip, 2015 [139]	Tissue	Progression	Let-7c
Zhang, 2011 [100]	Serum	Progression	miR-21
Yaman Agaoglu, 2011 [54]	Plasma	Progression	miR-21, miR-221
Shen, 2012 [56]	Plasma	Progression	miR-20a, miR-21, miR-145, miR-221
Watahiki, 2013 [123]	Plasma	Progression	miR-21, −126, −141, −151-3p, −152, −200c,-375, −423-3p
Nguyen, 2013 [116]	Serum	Progression	miR-141, miR-375, miR-378, miR-409-3p
Diagnostic/Progression			
Hao, 2011 [95]	Tissue	Diagnostic/Progression	miR-21
Mihelich, 2011 [98]	Tissue	Diagnostic/Progression	miR-96, miR-182, miR-183
Bryant 2012 [117]	Serum/Urine	Diagnostic/Progression	miR-181a-2, miR-625, miR-107, miR-574-3p, miR-20a, miR-23a, miR-624
Mavridis, 2013 [109]	Tissue	Diagnostic/Progression	miR-224
Larne, 2013 [108]	Tissue	Diagnostic/Progression	miR-96-5p, miR-183-5p, miR-145-5p, miR-221-5p
Walter, 2013 [110]	Tissue	Diagnostic/Progression	Up: miR-122, miR-335, miR-184, miR-193, miR-34, miR-138, Down: miR-373, −9, −198, −144 -215, −96, −222, −148, −92, −27, −125, −126, −27
Casanova-Salas, 2014 [111]	Tissue/Serum	Diagnostic/Progression	miR-187 and miR-182
Mihelich, 2015 [125]	Serum	Diagnostic/Progression	let-7a, −24, −26b, −30c, −93, −100, −103, −106a, −107, −130b, −146a, −223, −451, −874,
Kristensen, 2016 [112]	Tissue	Diagnostic/Progression	miR185-5p, miR-221-3p, miR-326
Treatment response			
He, 2013 [134]	Tissue	Treatment Response	miR-23b, miR-220, miR-221, miR-222, and miR-205
Lichner, 2013 [135]	Tissue	Treatment Response	miR-152
Gonzales, 2011 [133]	Plasma	Treatment Response	miR-141
Cheng, 2013 [120]	Serum	Treatment Response	miR-210
Someya, 2015 [136]	Blood	Treatment Response	miR-99a

**Table 2:** Summary of miRNA expression studies on prostate tumor/serum samples

miRNA investigated	Expression	Tissue	Reference
let-7a-2,let-7i,16-1,17-5p,20a,21,24-1,25,27a,29a,29b-2,30c,32, 34a,92-2,93-1,95,101-1, 106a, 124a-1,126a-1,135-2,146,149, 181b-1,184,187,191,196-1 197,199a-1, 214,128a, 195,198, 199a-1,199a-2,203,206,214,218-2,223,	up	tumor	Volina, 2006 [45]
202,210,296,320,370,373*,498,503	up	tumor	Porkka, 2007 [87]
let-7i,25,26a-1/2,31,32,34b,92-1/2,93,99b,106b,125a,181a-1/2,182,188,194-1/2,196a/2,200c, 370, 375,425,449	up	tumor	Ambs, 2008 [40]
221,222	up	tumor	Mercatelli, 2008 [42]
16,19b,24,100,125b,141,143,296	up	serum	Mitchell, 2008 [35]
141,200b, 200c	up	prostate cells	Mitchell, 2008 [35]
221	up	PCa cells	Mercatelli, 2008 [42]
221	up	tumor	Spahn, 2009 [91]
9*,15a,16,145,221,222,331,551a	up	PCa cells	Wang, 2009 [90]
128	up	PCa cells	Khan, 2010 [88]
let-7a,let-7c,let-7f,15b,20a,21,25,26b,30b,106a, 106b, 126*,148a,200c,218,375,	up	tumor	Szczyrba, 2010 [61]
25,93,106b	up	tumor	Poliseno, 2010 [61]
96,182,182*,183	up	tumor	Schaefer, 2010 [81]
141	up	plasma	Gonzales, 2011 [133]
182	up	Prostate cells	Mihelich, 2011 [98]
100,145,191,let7c	up	tumor	Leite, 2011 [138]
9*,141,200b,375,516a-3p	up	serum	Brase, 2011 [115]
21	up	plasma	Yaman Agaoglu, 2011 [54]
34c	up	PCa cells	Hao, 2011 [95]
20a,21,221	up	plasma	Shen, 2012 [56]
107,141,301a,326,432,484,574-3p,625*,2110	up	plasma	Bryant, 2012 [117]
21	up	tumor	Li, 2012 [55]
let-7b,7,9*,17,19b,20a,21,25,30d,32,92a-1*,93,95,96,106a*,106b, 106b*,130b,142-3p,141*, 148a, 153,182,182*,183,183*,200b*, 200c*, 210,301b,363,375,425,512-3p,583,615-3p,663,801,	up	tumor	Martens-Uzunova, 2012 [48]
let-7a,20a,21,106a,106b,375,	up	tumor	Wach, 2012 [10]
96,124,141,302b,375,378*,489,520d-5p,548a-3p,548c-3p, 875-5p,892b,	up	serum	Nguyen, 2013 [116]
96-5p,183-3p,183-5p	up	tumor	Larne, 2013 [108]
10b,15a,15b,16,18a,18b,20b,25,30c,32,34a,34c-5p, 92a, 122, 124,125a-5p,125b,128a,133b, 134, 135b,146b-5p,148b,181a,181b,181c,184,193a-5p,193b,206,214, 215,301a,372	up	tumor	Walter, 2013 [110]
19a,188-5p,574-5p,663,939,1224-5p,1225-5p,1249,1915,K12-3,UL70-3p,	up	tumor	He, 2013 [134]
21	up	tumor	Amankwah, 2013 [102]
32,106b-25,375	up	tumor	Hudson, 2013 [53]
96-5p,182-5p,183-5p,	up	tumor	Tsuchiyama, 2013 [106]
141,200a,200c,210,375	up	serum	Cheng, 2013 [120]
19a,19b,20a,20b,26a,26b,29c,106a,125a,125b,135a, 141, 148a,151–5p,174b,193a, 196b, 331-3p,365, 374a, 374b, 1274b,	up	tumor	Lichner, 2013 [135]
19b	up	tumor	Wang, 2014 [124]
182,SNORD78,U17b,U78_s,U78_x	up	tumor	Casanova-Salas 2014 [111]
17*,200b*,210,297,375,501-3p,551b,562	up	serum	Haldrup, 2014 [113]
345,519c-5p	up	serum	Wang, 2014 [124]
93,106b-25	up	PCa cells	Liang, 2014 [61]
Let-7a,103,107,130b,106a,26b,451,223,93,24,30c,874,100, 146a	up	serum	Mihelich, 2015 [125]
let-7a-5p,let-7d-3p,let-7d-5p,7-5p,7b-5p,20a-5p,21-3p,25-3p, 29b-2-5p,30d-3p,92a-3p, 92b-3p, 93-3p,96-5p,103a-3p,107, 130b-3p, 182-5p,183-5p,191-5p,196b-5p,200b-3p, 200b-5p, 200c-3p 329, 331-3p,339-3p,342-5p,375,421,423-3p,423-5p, 425-5p, 484, 615-3p,663a,663b,664a-3p, 1248,1260a	up	tumor	Kristensen, 2016 [112]
Let-7a,let-7b,let-7c,let-7d,let-7 g,16,23a,23b,26a,92,99a,103,125a,125b,143,145,195,199a, 199a*,221,222,497	down	tumor	Porkka, 2007 [87]
let-7b,1-2,34a,145,7-1/2,126,128a,133a-1,145,205,218-2,220,221,329,340,345,410, 487,490,494,499,520 h	down	tumor	Ambs, 2008 [40]
let-7b-g, 26a-b,29a-c,30a-e,99a-b,125a-b,145, 200a-b	down	tumor	Ozen, 2008 [79]
22,24,27a,27b,29a,30e,101,125a-5p,125b,143, 145, 152, 199a-5p,221,223,320,424,	down	tumor	Szczyrba, 2010 [61]
16,31,125b,145,149,181b,184,205,221,222	down	tumor	Schaefer, 2010 [81]
34c	down	tumor	Hagman, 2010 [92]
34c	down	PCa cells	Hao, 2011 [95]
30c,100,223,346	down	Prostate cells	Mihelich, 2011 [98]
141	down	plasma	Yaman Agaoglu, 2011 [54]
143,145,221	down	tumor	Wach, 2012 [10]
1,15a,16-1,133a	down	tumor	Kojima, 2012 [52]
1,10b,27b,29a,29b,34b,126,126*,130a,133a,133b,139-5p,142-3p,142-5p,143,143*,145,145*, 146a,149,150,155,181b,193b,193b*,204,205,221,221*,222,223,224,328,338-3p, 342-5p, 361-3p 378,378*,455-3p,455-5p,483-3p,485-3p,551b	down	tumor	Martens-Uzunova, 2012 [48]
181a-2*	down	plasma	Bryant, 2012 [117]
31-5p,34c-5p,205-5p,221-3p,222-3p	down	tumor	Tsuchiyama, 2013 [106]
101	down	tumor	Hudson, 2013 [49]
221,222	down	tumor	Amankwah, 2013 [102]
23b,26b,30c,155,181d,193a-5p,200b-5p,205,221,221-5p,222,224,335,374a,374b, 455-3p,505	down	tumor	He, 2013 [134]
145-5p,205-5p,221-5p,409-5p	down	tumor	Larne, 2013 [108]
224	down	tumor	Mavridis, 2013 [109]
222	down	serum	Cheng, 2013 [120]
409-3p,623,	down	serum	Nguyen, 2013 [116]
19a,19b	down	serum	Wang, 2014 [124]
34a*,34c-3p,187,221*,221,224,	down	tumor	Casanova-Salas 2014 [111]
1,133b	down	tumor	Karatas, 2014 [55]
1	down	Prostate cells	Chang, 2015 [46]
15a-5p,16-5p,19b-3p,22-3p,23a-3p,23b-3p,24-3p,26a-5p, 27a-3p, 27b-3p,29a-3p, 29b-3p,30a-3p,30a-5p,30c-5p,30e-3p, 30e-5p, 33a-5p,34a-5p,99a-3p,99a-5p,101-3p, 125b-2-3p, 125b-5p, 127-3p,130a-3p,132-3p,136-5p,143-3p,149-5p, 152, 154-5p,155-5p, 181a-5p,181b-5p,195-5p, 199a-3p,199a-5p, 199b-5p,205-5p, 214-5p,218-5p,221-3p,222-3p, 223-3p,335-5p,338-3p,362-3p,363-3p376a-3p,376c-3p,424-5p, 451a,455-3p, 455-5p,497-5p,502-3p,660-5p	down	tumor	Kristensen, 2016 [112]

### Diagnosis/progression of prostate cancer based on tissue

MicroRNA profiling in PCa tissues shows different expression patterns as compared with normal tissues. These unique miRNA expression profiling may provide tools for better prediction of diagnosis and progression of PCa. These data also assist to determine treatment strategies of PCa.

Porkka et al. [87] reported that differential expression of 51 miRNAs between BPH and prostate tumor tissues based on data from oligonucleotide array hybridization. Eight of these miRNAs were upregulated, while 22 were down-regulated in tumor tissues as compared with ones in BPH tissues. The authors observed an expression of several miRNAs, Let-7f, miR-19b, −184, and −198, were associated with advanced disease. Ambs et al. [40] evaluated mRNAs profiles detected in 60 LCM prostate tumors and 16 non-tumor prostate tissues using genome-wide expression assays. Expression of some miRNAs was significantly different comparing prostate tumors and non-tumor samples (miR-32, −133a-1, −490, −494, −520 h) as well as patients with organ-confined PCa vs. extra prostatic disease extension (miR-101-1/2, −200a, and -200b). These data suggested that miRNA dysregulation was related to development and progression of PCa. Further, the mRNA and miRNA analyses revealed influence the expression of cancer related genes by miRNAs in PCa cells. For example, the miR-106b-25 cluster maps to intron 13 of MCM7, which was significantly up-regulated in prostate tumors [40].

Ozen et al. [79] analyzed the expression of 480 human miRNAs between 10 benign peripheral zone tissues and 16 prostate tumors using microarrays and observed and validated down-regulation of several miRNAs including let-7c, miR-125b, and miR-145 in localized prostate tumor samples compared to benign peripheral zone tissue, and demonstrated miR-145 expression has a heterogeneous pattern in PCa tissue [79]. Mercateli et al. [42] showed overexpression of miR-221 is sufficient to strongly induce growth of LNCaP xenografts in mice. The tumor growth advantage in miR-221 expressing was significantly higher than mice in the referent group (*p* = 0.01). The average volume fold increase of miR-221 expressing tumors was higher than control tumors (*p* = 0.025) [42].

With high-throughput liquid phase hybridization (mirMASA) reactions and 114 miRNA probes, Tong et al. [76] observed downregulation of miR-221 and other miRNAs (miR-23b, −100, −145, and −222) in malignant prostate tissue (*n* = 40). They developed the 48 miRNA signature (including miR-135b and miR-194) that predicted biochemical recurrence after prostatectomy. Further evidence was provided for the growth modulatory roles of miRNAs by showing reduced ectopic expression of miR-23b, −145, −221 and −222 in LNCaP cell [76]. Khan et al. [88] analyzed over 1,000 proteins with multidimensional liquid phase peptide fractionation followed by tandem mass spectrometry, using 15 tissues samples, 5 of the prostate tumor, 5 of adjacent benign tissue and 5 of distant metastatic tissues. The study revealed the involvement of miR-128-a/b in regulating progression of PCa, where the miR-128 levels were elevated in lines of BPH prostatic epithelial cells, when compared to invasive PCa cells. The findings were validated in an independent set of 15 clinical specimens. Furthermore, transient overexpression of miR-128 in PCa cell lines attenuated cellular invasion, which suggest miR-128 as an important negative regulator of cellular invasiveness in vitro [88].

Prueitt et al. [80] showed a role of miRNAs in PCa progression by demonstrating a higher expression of 19 miRNAs (particularly miR-224) and downregulation of 34 miRNAs (particularly metallothioneins and proteins with mitochondrial localization and involvement in cell metabolism) in patients with progression than those with a locally confined disease [80]. However, Kristensen et al. [89] reported epigenetic downregulation of miR-224 and miR-452 (which are located in an intron of the GABRE) was a significant independent adverse predictor for time to BCR after radical prostatectomy in two large PCa patient cohorts (*n* = 293 and 198, respectively) [89]. Wang et al. [90] analyzed about 273 miRNAs using cell lines from 62 PCa patients with aggressive phenotype (Gleason score ≥ 8) and 63 PCa patients with nonaggressive phenotype (Gleason grade ≤ 5) and identified a significant association with PCa grade in 7 miRNAs included miR--16, −9*, −145, 222, −221, −331-3p, and -551a, especially the miR-145 and miR-331-3p, showed significantly down-regulated in aggressive PCa [90].

Schaefer et al. [81] analyzed tumor tissue and normal adjacent tissue from two groups of 76 and 79 men with untreated PCa. This study demonstrated miR-130b with a significantly different expression between normal and tumor tissues. miRs-125b, −205 and −222 were significantly related to tumor stage while miRs-31, −96 and −205 showed a significant correlations with Gleason score. When the combination of six miRNAs (miR-96, −149, −181b, −182, −205 and −375) was used, the AUC was significant (0.88) in discriminating normal and tumor tissue [81]. Spahn et al. [91] analyzed samples from BPH, primary PCa of a high risk group and corresponding metastatic tissues by microarray analysis. A PCa specific miRNA expression signature was generated based on the differential expression of 66 (48 downregulated and 18 upregulated) miRNAs between tumor and BPH tissues (*p* < 0.01). They observed a progressive downregulation of miR-221 in aggressive and metastatic PCa. Downregulation of miR-221 was also associated with Gleason score and clinical recurrence [91].

Hagman et al. [92] found the relative expression of miR-34c was significantly lower in the PCa samples compared to the BPH samples (*p* = 0.0005) and decreased with higher grade. The miR-34c level in patients with metastasis was found to be significantly lower than patients without metastasis (*p* = 0.02). The expression of miR-34c distinguishes aggressive from non-aggressive PCa (*p* = 0.012) and was inversely correlated with PSA level. Although there was no significant correlation between miR-34c expression levels and treatment/response to treatment, the expression of miR-34c was significantly correlated with survival [92].

Pesta et al. [93] evaluated the expression of four miRNAs, miR- let-7a, −15a, −16, and 20a in 53 prostate tumor and 85 benign prostatic hyperplasia (BPH) tissues to investigate whether miRNA expression is related to clinic-pathological features of PCa. They observed a difference in expression for miR-20a in patients with a Gleason score of 7–10 compared with patients with a low Gleason score of less than 6, suggesting an oncogenic role for miR-20a in PCa development [93]. Although the previous study demonstrated the potential of miR-20a to distinguish between more and less aggressive PCa disease, the use of BPH tissue is not the most appropriate control relative to normal tissue and potentially impede the identification of unique miRNAs in PCa. On the other hand, Taylor et al. [94] reported that, the copy-number alterations in prostate tumors modestly revealed distinct subgroups with substantial differences in time to biochemical recurrence while unsupervised hierarchical clustering of mRNA and miRNA data failed to identify robust clusters of patients with significant differences in prognosis [94].

Szczyrba et al. [61] found 16 miRNAs upregulated by at least 1.5-fold and 17 miRNAs downregulated by at least 1.5-fold in snap frozen prostate tumor tissue. Strong upregulation was found for miR-148a, −200c, −375, and the miR-143, −145 and −223 were downregulated [61]. In another study by Hao et al. [95], miR-21, and miR-141 were upregulated at least 2-fold, while miR-10, −16, −34c, and -125b, were downregulated in formalin fixed paraffin embedded (FFPE) tumor tissues as compared with BPH specimens [95]. The positive predictive value was enhanced from 40 to 87.5% when the PSA variable was added with expression variable of miR-21 and miR-141. After analysis of tumor samples from Caucasians and African-Americans, miR-182 level tended to be higher in PCa tissues and miR-30c, −100, −223, and −346 were expressed at lower levels in PCa tissue in Caucasians.

Therefore, miR-21 may indicate a potential role of carcinogenesis in prostate tissue [57]. In addition, multiple studies reported that miR-21 is an oncogene in prostate cancer and over-expression may be related to the development of castration-resistance PCa [57–60, 96]. In addition, Bonci et al. [97] recently reported over-expression in PCa metastasis.

A RNA microarray profiling analysis of a patient cohort with organ-confined prostate tumors indicated the overexpression of miR-96, −182, and −183 with at least 2-fold in laser captured micro-dissected (LCM) PCa tissue compared with normal adjacent prostate tissue [98]. Moltzahn et al. [99] identified miRNA signatures which can distinguish healthy controls from patients and correlate with a prognosis. Cluster and ROC analyses indicated the diagnostic potential and the ability to further separate PCa patients according to their risk of recurrence by their miRNA signatures [99]. Zhang et al. [100] assessed a potential role of serum miR-21 in the progression of PCa with 56 patients, including 20 localized PCa, 20 with androgen-dependent PCa (ADPC), 10 with hormone-refractory PCa (HRPC), and 6 with BPH. Significantly higher levels of miR-21 were detected in patients with ADPC and HRPC, especially in those resistant to docetaxel-based chemotherapy. These findings suggest miR-21 may be a biomarker at the transition to hormone refractory disease and a potential indicator for response to treatment in PCa patients [100].

Martens-Uzunova et al. [48] analyzed the expression of miRNAs in 102 prostate tumor tissues, adjacent normal tissues, lymph and trans-urethral resection from normal and PCa patients. Regarding the miRNAs, the authors analyzed 80 miRNA species, where 22 were identified with significant differences between the two groups with or without PCa that can serve as a diagnosis markers. The authors also identified a miR profile consist of 25 miRNAs, which 12 overexpressed and 13 under-expressed in prostate tumor tissues with poor outcome. This 25 miRNA profile is very accurate to predict a recurrence (AUC = 0.991, *p* < 0.0001 Kaplan-Meier analysis). They also observed that the overall expression of miRNA was lower in PCa malignant lymphoma compared to the PCa prostate tissues. 19 miRNAs have been upregulated in metastatic lymph node while 69 miRNAs, including miR-1, −133a, −133b, −143, −143*, −145, −145*, −204, −221 and −222, have been downregulated in comparison to PCa tissue [48].

Li et al. [55] confirmed the miR-21 expression in 169 PCa tissue samples was significantly associated with clinical variables, such as stage, lymph node involvement, capsular invasion, organ confined disease, Gleason score, BCR and patient follow-up. The relationship between miR-21 as BCR persisted even after adjusting for standard clinico-pathological parameters (i.e., PSA, patient age, Gleason score, surgical margin, lymph node metastasis, capsular invasion, and pathological stage) [55].

Avgeris et al. [101] evaluated the clinical utility of miR-145 using 137 prostate tumor tissues and 64 benign samples. They found that the reduction of miR-145 expression in PCa was correlated with Gleason score, clinical stage, tumor size and PSA level and follow-up PSA levels. Further, the reduction of miR-145 expression was significantly associated with increased risk for biochemical recurrence and shorter disease-free survival (DFS) among PCa patients. This relationship remained significant after adjusting for Gleason score, clinical stage, PSA and age at diagnosis [101]. We [102], reported a significantly increased risk for recurrence (*p* < 0.0001) in patients with decreased expression of miR-21, but not for miR-221 (*p* = 0.57) or miR-222 (*p* = 0.24). An increased risk of recurrence was more prominent in obese (*p* = 0.031) than non-obese patients. After adjustment for stage and Gleason score, the results showed miR-21 expression was independently linked to disease recurrence among obese patients [102].

Barnabas et al. [103] observed a copy number gain of miR-151 gene at 8q24 in PCa tissues from African-American men who developed BCR after radical prostatectomy. These data suggested a copy number gain of miR-151 gene in primary tumor that may indicate the presence of metastatic disease [103]. Leite et al. [104] observed a 2.7-3.4 fold higher likelihood of BCR when miR-100, −145, −191, and let-7c were over-expressed. However, only miR-100 showed a significant association independently with BCR in multi-variable analysis [104].

Srivastava et al. [105] evaluated expression of 8 miRNAs in 40 paired prostate tissues. miR-205, miR-214, miR-221 and miR-99b were significantly downregulated in PCa tumor tissue. These miRNA profiles can discriminate PCa patients from healthy individuals successfully [105]. Tsuchiyama et al. [106] tested whether levels of miRNAs were correlated with aggressiveness. Expression of miR-315p, −34c-5p, and −205-5p was significantly decreased compared to those in normal tissues regardless of Gleason pattern (*p* < 0.05). Meanwhile, in the same patients with high grade PCa, expression of miR-31-5p −182-5p, and −205-5p in tumor tissues was higher than ones obtained from intermediate Gleason tumor (*p* < 0.05) [106]. Using prostate tissues from 23 PCa and 25 BPH patients from Turkey, 4 candidate miRNAs were evaluated (i.e., miR-133b, −203, −221, and −361-3p). miR-361-3p was significantly lower in tumor tissues from PCa than BPH patients (*p* = 0.004). Both miR-133b and miR-221 were downregulated in PCa compared to BPH (*p* < 0.01 and 0.03, respectively). The levels of miR-203 were found to be significantly upregulated in PCa samples (*p* = 0.0002). The AUC values of all miRNAs, miR-133b, miR-203, miR-221, and miR-361-3p were 0.73, 0.81, 0.71, and 0.74, respectively [107].

Larne et al. [108] developed a formula which can predict early stage of PCa with aggressive progression characteristics. Using differentially expressed miRNAs in PCa tissue samples, the authors developed a miRNA signature, consisting of four miRNAs [(miR-96-5p x miR-183-5p)/(miR-145-5p x miR221-5p)], denoted as the miRNA index quote (miQ). The miQ provided an excellent diagnostic discrimination. The median of miQ is 17 times higher in the PCa group compared to the non-PCa group (*p* < 0.0001). Further, miQ level is also associated with grades (*p* = 0.0067), PSA levels (*p* < 0.0001), metastasis (*p* < 0.0001) and survival (*p* = 0.0014). More importantly, miQ distinguishes aggressive tumors from non-aggressive PCa (*p* < 0.0001) with an AUC of 0.90 (95% CI: 0.77–0.97) [108].

Mavridis et al. [109] reported that miR-224 could distinguish PCa patients from BPH patients with an AUC of 0.67 (*p* = 0.001) in tissue samples. miR-224 expression inversely correlated with aggressiveness (*p =* 0.017), thus Gleason score, advanced disease stage, PSA, risk of relapse and positively with progression-free survival [109]. Walter et al. [110] found upregulation of miR-143 and miR-146b and loss of 18 miRNAs (e.g. miR-34c, −29b, −212 and -10b) in the PCa tumors relative to normal epithelium and/or stroma (*p* < 0.001). In addition, upregulation of 11 miRNAs and downregulation of 7 miRNAs were found in the tumors with high Gleason score (≥8) when compared with ones with Gleason score 6 [110]. Casanova-Salas et al. [111] found the expression miR-182 was increased in PCa tissue samples and decreased 6 miRNAs, miR-34a*, −34c-3p, −187, −221*, −221 and. 224. Further miR-182 and miR-187 were also differentially expressed according to clinical variables, such as Gleason score, stage, status of TMPRSS2-ERG and progression [111].

In one of the largest miRNA profiling studies published for PCa, Kristensen et al. [112] analyzed the expression of 752 miRNAs in a training set of 13 nonmalignant and 134 PCa tissue samples. Subsequently, a total of 93 miRNAs with diagnostic/prognostic potential were selected for further validation in two similar-sized independent patient cohorts. The authors identified several novel deregulated miRNAs in PCa as compared to nonmalignant prostate tissue samples as well as between more/less aggressive PCa subgroups, as defined by clinic-pathological parameters. Most notably, the authors also identified a novel 3-miRNA prognostic signature (miR-185-5p + miR-221-3p + miR-326) predicted time to biochemical recurrence after radical prostatectomy independently of routine clinical variables in a training cohort as well as in two independent validation cohorts. This is the first report to demonstrate independent prognostic value for a miRNA signature in three independent PCa patient cohorts, supporting the potential clinical relevance of this signature [112].

### Diagnosis/progression of prostate cancer based on serum

Some studies have examined the differential expression of miRNAs in serum or plasma in an attempt to develop a blood based diagnosis, detection of progression and recurrence of PCa. Mitchell et al. [35] showed that serum levels of miR-141 can distinguish PCa patients from healthy controls, supporting the potential role of this miRNA as a diagnostic marker for PCa. The up-regulation of miR-141 in PCa patients was confirmed in later studies [54, 95, 113–117]. Mahn et al. [118] found circulating oncogenic miRNAs (i.e., miR-26a, −32, −195 and let-7i), particularly miR-26a have a sensitivity of 89% and specificity of 56% (area under the curve: AUC = 0.70) for diagnosis. The diagnostic accuracy increased when these four miRNAs were analyzed in combination (sensitivity: 78.4%, specificity: 66.7%, AUC = 076). Increased levels of miR-16, −26a, and −195 were inversely associated with surgical margin positivity. miR-195 and -let7i were also inversely associated with Gleason score. They found a significant reduction of miR-16, −26a, and −195 levels after radical prostatectomy. miR-32 was found to be down-regulated in PCa tissue (*p* = 0.02) [118].

Chen et al. [119] found that a panel of five serum miRNAs (let-7c, let-7e, miR-30c, −622 and −1285) were significantly different in PCa patients as compared to healthy controls in both identification and validation cohorts. All 5 miRNAs could distinguish PCa from healthy controls individually. The respective sensitivity and specificity values were: let-7c, 68.5 and 70%; let-7e, 77.8 and 75%; miR-30c, 79.6 and 68.8%; miR- 622, 90 and 63.0%; miR-1285, 61.3 and 57.4% [119]. Wach et al. [10] reported on the detection of miR-145 in endothelial cells of blood vessels but not stromal cells. Based upon the ratio of miRNA expression, they concluded that miR-145 may be the best negative predictive biomarker suitable for diagnostic or prognostic purposes in PCa [10].

Selth et al. [114] used a mouse model of PCa as a tool to discover serum miRNAs that could be used in a clinical setting. Among 45 miRNAs identified, four miRNAs (miR-141, −298, −346, and −375) were significantly elevated in metastatic PCa samples as compared to those in healthy controls (all *p* < 0.05). miR-141 and miR-375 were associated with biochemical recurrence (BCR) [114]. Cheng et al. [120] identified five serum miRNAs (miR-141, −200a, −200c, −210, and −375) associated with metastatic castration resistant PCa in both screening and validation cohorts. In the screening cohort, serum levels of five miRNAs were significantly increased in PCa cases as compared with controls (miR-141: *p* < 0.0001, AUC = 0.90; miR-200a: *p* = 0.007, AUC = 0.70; miR-200c: *p* = 0.017, AUC = 0.72; miR-210: *p* = 0.02, AUC = 0.68; and miR-375: *p* = 0.009, AUC = 0.77), while the respective p-values of these five miRNAs were 0.001, 0.073, 0.055, 0.022, and 0.021 in a validation cohort [120].

Haldrup et al. [113] performed genome-wide miRNA profiling of serum samples from 13 BPH control and 31 PCa patients. The dysregulation of miR-141 and −375, two of the most well-documented candidate miRNA markers for PCa, was confirmed. Further, they developed three novel and highly specific miRNA panels (miR-562/-210/-501-3p/-375/-551b) able to identify 84% of all PCa patients, 80% of patients with disseminated PCa (let-7a*/miR-210/-562/-616), and 75% of disseminated PCa patients when compared to localized PC patients (miR-375/-708/-1203/-200a) [113]. miR-141 levels were significantly increased in patients with a higher Gleason score (*p* = 0.05) although the levels of miR-26a-1 and −141 in 170 pre-biopsy serum samples were similar in patients with positive and negative biopsies (*p* = 0.72, AUC = 0.52 and *p* = 0.84, AUC = 0.49, respectively) [121].

The expression levels of 4 miRNAs in plasma from 59 PCa patients with different clinic-pathological characteristics and two groups of controls, 16 BPH samples and 11 young asymptomatic men (YAM) were analyzed to evaluate their diagnostic and prognostic value in comparison to PSA. miR-375 was significantly downregulated in 83.5% of patients compared to BPH controls and showed stronger diagnostic accuracy (AUC = 0.81, 95% CI: 0.70–0.92, *p* = 0.00016) compared with PSA (AUC = 0.71, 95% CI: 0.56–0.86, *p* = 0.013). Sensitivity of 86.8% and specificity of 81.8% were reached when all biomarkers were combined (AUC = 0.88) and YAM were used as calibrators [122].

In a study of 51 PCa patients and 20 healthy controls in Turkey, Yaman Agaoglu et al. [54] investigated the levels of miRs-21, −141, and −221 in plasma of patients and healthy controls. They found miR-21 and miR-221 levels were significantly higher in prostate patients than controls (*p* < 0.001, ROC-AUC = 88% and *p* < 0.001, AUC = 83%; respectively), but not for miR-141 (*p* = 0.20). miR-141, however, was found to be significantly higher in local advanced patients in comparison with those diagnosed with local outcome (*p* < 0.001, AUC = 76%) [54]. Brase et al. [115] observed enhanced expression of miR-375 and miR-141 in prostate tumor tissue compared to normal tissue and both miRNAs were upregulated in the sera of patients with metastatic disease compared to those with localized disease and correlated with high Gleason score or lymph-node positive status. These findings show miR-375 and miR-141 detection in serum may be associated with advanced disease [115].

Nguyen et al. [116] demonstrated over-expression miR-375, −378, and −141 were significantly in serum collected from castration resistant PCa patients compared to those with localized disease, while miR-409-3p was significantly under-expressed. In prostate tumor tissues, the expression of miR-375 and miR-141 were significantly higher than those in normal prostate tissue [116]. Relative to normal tissue, miR-205 (down-regulated in mCRPC) was indicated by Watahiki et al. [123] to be associated with a lower Gleason score and a lower probability of both BCR and clinically evident metastatic events after prostatectomy. To the contrary, miR-141, 151-3p, 152 and 423-3p are inversely associated with prognosis and/or Gleason score. miR-141 and −152 may identify individuals with a high probability of recurrence after surgical treatment. miR-423-3p and miR-205 were suggested as novel prognostic factors due to their correlation with several clinical parameters [123].

Wang et al. [124] investigated whether pre-surgical serum levels of miRNAs can identify PCa patients. miR-19a and miR-19b displayed higher expression in the case group compared to controls, consistent with higher serum levels in cases. Conversely, miR-345 and -519c-5p had significantly lower expression among PCa patient. These results were consistent with lower levels in the patients’ serum samples. Logistic regression models for predicting case/control status showed all four miRNAs as highly significant predictors of outcome status even when controlling for age, PSA, clinical stage, and degree of biopsy involvement. In the validation cohort, expression data of 4 miRNAs were consistent with data from the discovery cohort. Models consisting of a combination either miR-19a or -19b together with miR-345 and - 519c-5p showed an AUC of 0.94, reaching high significance (*p* = 0.02 and *p* = 0.017 respectively) [124].

Shen et al. [56] also found miR-20a, −21 and −145 levels were significantly associated with PCa but not disease aggressiveness. The combination of these three miRNAs was a significant predictor and distinguished high risk PCa patients [56]. Martens-Uzunova et al. [48] evaluated some miRNAs significantly different in lymphocytes that could be used in the evaluation of progression of PCa, although some demonstrated an upregulation 2 fold-change or more between normal and PCa tissue (miR- 95, 96, 32, 153,182, 182*, 183) were and others (miR-133a, 133b, 221, 221*, 222, 224, 338-3p, 378*, 455-5p) were downregulation, especially miR-205 showed 6.3 fold downregulation [48]. Karatas et al., studying tissue samples from patients with recurrent and non-recurrent PCa, confirmed a negative uptake of miR-1 and miR-133b, miR-145* in recurrent Pca [51].

Mihelich et al. [125] developed the equation, miR Risk Score based on 7 miRNAs, miR, let-7a, −26b, −106a, −107, −130b, −223, and −451 in plasma samples. This scoring system showed to be highly predictive of low-grade PCa. The AUC for low-grade PCa was 0.69 across the range of miR risk scores [125]. Bryant et al. [117] reported 12 miRNAs were differentially expressed in PCa patients plasma compared with controls. Among the 12 miRNAs, 11 were significantly correlated with metastases. The association of two miRNAs, miR-141 and miR-375 with metastatic PCa was confirmed in a separate cohort. An analysis of urine samples indicated miR-107 and miR-574-3p were significantly associated with PCa risk [117].

### Diagnosis/progression of prostate cancer based on urine and ejaculation

Srivastava et al. evaluated expression of 8 miRNAs in urine and tissue samples. miR-205, and miR-214 were significantly downregulated in PCa patients in both tissue and urine specimens. This miRNA profile can discriminate PCa patients from healthy individuals with 89% sensitivity and 80% specificity [105]. Haj-Ahmad et al. [126] performed miRNA expression profiling with urine samples obtained from 8 PCa patients, 12 BPH patients and 10 healthy males using whole genome expression analysis. Differential expression of two individual miRNAs between healthy males and BPH patients was detected and found to possibly target genes related to PCa development and progression among 894 miRNAs assayed. The authors found the sensitivity and specificity of miR-1825 for detecting PCa among BPH individuals were 60 and 69%, respectively. Further, the sensitivity and specificity for miR-1825 and −484 using tandem deregulation at the same time were 45 and 75%, respectively [126]. Another urine study suggested miR-483-5p was significantly associated with PCa based on samples from 71 PCa patients and 18 controls (*p* = 0.01; AUC = 0.69) [127].

Roberts et al. [128] evaluated the diagnostic performance of ejaculated-derived PCA3, Hepsin, and miRNAs (miR-125b, −200b, −200c, and −375) in 61 PCa patients. In stratified analysis, miR-200c (AUC = 0.79) and miR-375 (AUC = 0.76) demonstrated as the best single marker performance, while a combination of serum PSA, miR-200c and miR-125b further improved a prediction capacity for PCa status as compared to PSA alone determined by biopsy (AUC = 0.87 vs. 0.67; *p* < 0.05), and prostatectomy pathology (AUC = 0.81 vs. 0.70). For PCa status by biopsy, the specificity enhanced from 11% for PSA only to 67% for a combination of PSA, miR-200c, and miR-125b, with 90% specificity [128].

### miRNA as predictors of therapeutic response for prostate cancer

miRNAs affect the expression of multiple genes in disease pathways and therefore represent interesting drug targets. Different experimental approaches used to validate the function of miRNAs can be potentially developed for miRNA-based therapies. For example, over-expressed oncogenic miRNAs can efficiently be inhibited using modified antisense oligonucleotides and this strategy was positively evaluated in animal models [129–131]. Expression of down-regulated tumor suppressor miRNA can be restored using synthetic precursor double stranded miRNA molecules or miRNA expression vectors delivered through lentiviral systems [67, 86]. miRNAs can also be exploited to increase the sensitivity of tumor cells to conventional anticancer agents [86]. For example, identification of miRNAs involved in the transition from androgen-independent to androgen-dependent state will aid in the modulation of the androgen-independent phenotype with the aim of increasing the responsiveness of advanced PCa to anticancer therapies. Some studies have currently demonstrated miRNAs are differentially expressed in androgen-independent compared to androgen-dependent PCa cell lines [65, 66, 73, 132].

With Low-Density Arrays (TLDA) to screen differentially expressed serum miRNAs, Cheng et al. [120] found that serum level of miR-210 were correlated with PSA level change per day during treatment (*p* = 0.029). Furthermore, expression of miR-210 was significantly lower in non-aggressive disease patients, as compared to those with recurrence (*p* = 0.001). The authors suggest that a subset of mCRPC patients may have a higher hypoxia response signaling, leading to increased miR-210 and therapy resistance. These data suggested miR-210 levels could be used to identify a distinct, subset of mCRPC patients with tumor-associated hypoxia. Gonzales et al. [133] studied in 21 PCa patients in a longitudinal manner with multiple blood draws. They determined the levels of miR-141 in PCa patients and compared it to the levels of three other conventional PCa biomarkers: PSA, lactate dehydrogenase (LDH) and circulating tumor cells (CTC). Then they found that miR-141 level changes were similar with changes observed by PSA, LDH and CTC and to the clinical assessments of the patients, suggests miR-141 can be used as a marker for therapeutic response in PCa patients. He et al. [134] reported that 28 miRNAs were differentially expressed between PCa tumor and adjacent benign tissues. The authors compared the miRNA expression profiles to non-Chinese populations and found that miR-23b, −220, −221, −222, and −205 may be common targets for treatments in all populations. This study also identified 15 specific miRNAs in Chinese patients. miR-374b and miR-19a showed significant correlations with clinical-pathological features in Chinese patients [134].

Lichnew et al. [135] confirmed results for 2 miRNAs were downregulation of miR-331-3p (*p =* 0.009) in the low-risk BCR group and miR-152 in the high-risk group (*p =* 0.012). To further confirm their results, using a second independent set of PCa cases, they observed similar results, as expected. The prediction model correctly classified high-risk individuals (33/35, 94.3%) and low-risk individuals (6/29, 20.7%) [135]. Someya et al. [136] investigated an association between miRNA expression and radio-sensitivity of PCa patients. They observed different miRNA expression among PCa patients with different radiation treatments. Three miRNAs (miR-99a, −147 and −508) in non-irradiated peripheral blood lymphocytes (PBLs) and one miRNA in irradiated PBLs (miR-199b), and significant induction of 11 miRNAs by irradiation (miR- 28, −185, −221, −340, −376c, −422a, −486, −491, −542, −652 and −660) were found. The authors concluded a combination of low ATP-dependent DNA helicase II (Ku80/XRCC5) expression and highly-induced miR-99a expression could be a promising marker for predicting radio-sensitivity after radiotherapy [136].

## Conclusions

miRNAs regulate gene expression post-transcriptionally by both destabilizing mRNA and inhibiting their translation. Post-transcriptional regulation guarantees rapid and reversible changes in protein synthesis without altering transcription. Further, miRNAs can simultaneously modulate several cancer-relevant gene networks, because of their ability to bind to several targets. These attributes make miRNAs attractive candidates relevant as one-hit multi-target (polypharmacological) therapeutic agents against PCa and cancer in general. Further, miRNAs represent a potential source of PCa diagnostic biomarkers. The stability of miRNA in serum and plasma and previous work showing differential expression of serum miRNA in normal and tumor tissue suggest the potential of miRNA as blood-based biomarkers for PCa diagnosis.

However, detailed knowledge of their expression, regulation, target genes and mechanism of action is required to achieve this goal. Moreover, it is critical to establish standard protocols for miRNA normalization and collection of tissue biospecimens. Some studies analyze miRNA expression in laser micro-dissected tumor tissue to procure tumor tissue with high purity [40, 98, 105, 106]. Unfortunately, other studies continue to analyze miRNA expression in snap frozen or FFPE tissue as observed in this review [61, 95, 101]. RNA endogenous controls (e.g., RNU44, RNU6B) can vary from study to study. The analysis of miRNA expression in non-micro-dissected tumor tissue and use of various miRNA endogenous controls, may skew miRNA expression profiles in PCa. For biological fluids (i.e., serum, plasma, urine), there is no designated endogenous control to normalization miRNA expression in cancer. However, some studies utilize stable synthetic Caenorhabditis elegans (i.e., Cel-39, −54, −238) as spike-in internal controls to normalized miRNA expression in serum [35, 115]. It is well known that African American men are 2.5 times more likely to die from PCa relative to Caucasian men [1]. In this review, majority of the miRNA profiling studies utilized pre-dominantly Caucasian populations. Given this apparent racial disparity in PCa, more miRNA profiling studies in diverse populations are needed to identify ethnic-specific miRNAs that may possibly play a role in aggressive disease among African American men. Ultimately, miRNA profiling in tumor tissue and biological fluids collected from more diverse populations using more standardized miRNA normalization protocols may lead to the identification of more key miRNAs and elucidate the role of miRNAs in PCa disease prevalence in certain populations.

Further studies in in vivo knockout or knock-in models should be helpful in understanding the pathways affected by miRNAs. Further, gene expression and proteomic analysis associated with loss- and gain-of-function studies in different model systems might contribute to a better understanding of the cellular networks affected by miRNA in the development and progression of cancer [5]. Additional investigation is also warranted to ascertain whether dysregulation of miRNA expression is causative rather than a consequence of cancer.

In summary, expression profiling studies provide evidence for the role of miRNAs in PCa diagnosis and prognosis. However, limited overlap exists between these studies and an extremely limited number of PCa-related miRNAs are currently known. Present promising candidates are miR-1, −21, 106b, 141, miR-145, miR-205, miR-221, and miR-375, which are the most frequently studied and seem to be the most promising for diagnosis and prognosis for prostate cancer.
